# Investigating the spatial risk distribution of West Nile virus disease in birds and humans in southern Ontario from 2002 to 2005

**DOI:** 10.1186/1478-7954-5-3

**Published:** 2007-05-01

**Authors:** Heidi Beroll, Olaf Berke, Jeffrey Wilson, Ian K Barker

**Affiliations:** 1 Department of Population Medicine, Ontario Veterinary College, University of Guelph, Guelph, Ontario, Canada; 2 Department of Biometry, Epidemiology and Information Processing, School of Veterinary Medicine Hannover, Hannover, Germany; 3 Foodborne, Waterborne and Zoonotic Infections Division, Public Health Agency of Canada, Guelph and Ottawa, Ontario, Canada; 4 Canadian Cooperative Wildlife Health Centre, Ontario/Nunavut Region, Department of Pathobiology, Ontario Veterinary College, University of Guelph, Guelph, Ontario, Canada

## Abstract

**Background:**

The West Nile virus (WNv) became a veterinary public health concern in southern Ontario in 2001 and has continued to threaten public health. Wild bird mortality has been shown to be an indicator for tracking the geographic distribution of the WNv. The purpose of this study was to investigate the latent risk distribution of WNv disease among dead birds and humans in southern Ontario and to compare the spatial risk patterns for the period 2002–2005. The relationship between the mortality fraction in birds and incidence rate in humans was also investigated.

**Methods:**

Choropleth maps were created to investigate the spatial variation in bird and human WNv risk for the public health units of southern Ontario. The data were smoothed by empirical Bayesian estimation before being mapped. Isopleth risk maps for both the bird and human data were created to identify high risk areas and to investigate the potential relationship between the WNv mortality fraction in birds and incidence rates in humans. This was carried out by the geostatistical prediction method of kriging. A Poisson regression analysis was used to model regional human WNv case counts as a function of the spatial coordinates in the east and north direction and the regional bird mortality fractions. The presence of disease clustering and the location of disease clusters were investigated by the spatial scan test.

**Results:**

The isopleth risk maps exhibited high risk areas that were relatively constant from year to year. There was an overlap in the bird and human high risk areas, which occurred in the central-west and south-west areas of southern Ontario. The annual WNv cause-specific mortality fractions in birds for 2002 to 2005 were 31.9, 22.0, 19.2 and 25.2 positive birds per 100 birds tested, respectively. The annual human WNv incidence rates for 2002 to 2005 were 2.21, 0.76, 0.13 and 2.10 human cases per 100,000 population, respectively. The relative risk of human WNv disease was 0.72 times lower for a public health unit that was 100 km north of another public health unit. The relative risk of human WNv disease increased by the factor 1.44 with every 10 positive birds per 100 tested. The scan statistic detected disease cluster in the bird and human data. The human clusters were not significant, when the analysis was conditioned on the bird data.

**Conclusion:**

The study indicates a significant relationship between the spatial pattern of WNv risk in humans and birds.

## Background

### West Nile Virus

West Nile virus (WNv) was first isolated and identified in 1937 from the blood of a resident of the West Nile district of Uganda [1,2]. Subsequently WNv caused outbreaks of human cases in Egypt, Israel, South Africa and in some parts of Europe and Asia [3]. WNv became a veterinary public health concern in North America in August of 1999, signaled by an outbreak in New York City. There are a number of theories on how the virus was able to survive and be transmitted during the spring of 2000. One is that infected mosquitoes from the 1999 New York outbreak were able to survive by hibernating through the winter in underground sewers, abandoned buildings, and bunkers [4]. Another implicates chronically infected migratory birds that may have reintroduced the virus after returning from the south the following spring [5].

The virus appeared in Canada in 2001, where the first mosquito and bird cases were recognized in Ontario in August [6]. The first human WNv cases in Canada occurred in Ontario and Quebec, in 2002 [6,7].

WNv is an emerging pathogen in Canada causing disease in animals and humans. As a *Flavivirus *of the Japanese encephalitis virus serogroup [8] WNv is maintained in an enzootic cycle involving viremic birds and ornithophilic mosquitoes, particularly *Culex *species. As a spill over effect WNv may be transmitted to humans or other dead-end hosts, if the mosquitoes change their host preference [9] or if a bridge vector is involved [10].

Birds are the most important reservoir host and are able to amplify the disease since they develop high-level viremia (increased quantity of virus that replicates and circulates within the blood of the host) and remain infectious for several days [4,5]. In North America, the virus has been found in more than 150 bird species. Of these, corvids are among the most susceptible to infection and comprise an auspicious component of the mortality [11,12].

Wild bird mortality has been investigated for tracking the geographic distribution of WN virus in North America [7,12,13]. During the outbreak in New York City in 1999, a large die-off of birds, especially corvids, was associated with the outbreak in humans, both spatially and temporally [14]. Reports of the extensive die-off of birds preceded the epidemic in humans for the majority of regions [15,16]. For example, Marfin et al. [15] found that in 2000 in Northeastern United States, all 21 infected individuals had an illness onset date that was at least 15 days after the date that WNv infected birds were first collected in the individual's county of residence. From this, it has been suggested that dead birds can provide an early warning system to help predict areas of high human risk [15]. Dead bird surveillance programs may allow prevention and control methods to be intensified, before an outbreak of human cases occurs. Dead bird surveillance data are commonly used in assessing WNv risk; however different modeling approaches have been explored.

A study by Johnson et al. [17] quantified the association between clusters of dead crow sightings and onset of human WNv case in New York State. The risk in humans was positively associated with living in towns in proximity to dead crow clusters.

Theophilides et al. [18] developed the dynamic continuous-area space-time system (DYCAST) to identify and monitor high risk areas for WNv infection. The Knox test was used to assess the significance of space-time interaction in dead bird reports as an indicator of an intense WNv amplification cycle. It successfully identified areas of high risk for human WNv infection in areas where five of seven human cases resided, at least 13 days prior to the onset of illness, in New York City in 2001.

A study by Eidson et al. [19] evaluated the usefulness of dead bird surveillance in New York City in 2000 for detecting the geographic spread of the WNv and for providing an early warning system for humans. This study found that a steep increase in the number of dead crow sightings predated the onset date for the first human case and the increase in WNv positive birds by several weeks.

Watson et al. [20] assessed the spatial relationship between the locations of dead crow sightings reported early in the transmission season and the residences of WNv infected individuals in Chicago in 2002. Smoothed dead crow density values generated using kernel estimation were reclassified into high and low crow mortality areas. This study identified a spatial association between early season crow deaths and WNv infected residences of Chicago. Among humans the crude rate for WNv infection was 10.8 cases per 100,000 inside the high crow mortality areas compared to 3.2 cases per 100,000 outside.

The aim of this study was to investigate the latent risk distribution of WNv disease among birds and humans in southern Ontario and to compare the spatial risk patterns for the period 2002–2005, with a relatively simple but robust analysis. The objectives were to (1) describe the spatial variation of crude rates and smoothed risk for WNv among the public health units (PHUs) of southern Ontario from 2002 to 2005, (2) describe the geographical risk distribution and variation of WNv disease in humans and tested dead birds in southern Ontario from 2002 to 2005 in the form of risk maps, (3) investigate the potential for the tested dead bird data to be used as an indicator of human WNv risk, (4) explore the bird and human data for disease clusters.

### Disease mapping

Spatial epidemiology is the description and analysis of geographic variations in disease with respect to demographic, environmental, behavioral and infectious risk factors [21]. In public health, identification and quantification of patterns in disease occurrence provide the first steps toward increased understanding and possibly, control of that particular disease [22]. In order to better understand the spatial epidemiology of the WNv, including the trends and clusters within the risk distribution, disease maps are essential.

Disease maps provide a rapid visual summary of geographic information and may identify subtle patterns in the data that are missed in tabular presentations. They are used variously for descriptive purposes, for surveillance to highlight areas at apparent high risk, to aid resource allocation [21], to identify possible disease clusters and to show changes in disease patterns over time [23].

There are various mapping techniques that can be used depending on the type of spatial data being analyzed. Dot or spot maps are used to visualize spatial point data. These maps indicate the location of case or event data on a geographical map, such as the location of WNv cases. Choropleth maps are used to explain spatial variation in regional count data, such as the number of WNv cases per PHU. These maps symbolize regional statistical data within the boundaries of geographic regions grouped into classes. Each class stands for a range of values and is represented by a logical sequence of gray (or color) tones.

When regional health information, such as the incidence rate of WNv at the public health unit level is considered as point measurement data at the regional centre (i.e. geostatistical data), then isopleth maps can visualize the spatial distribution of the latent risk. Isopleth maps show the distribution of spatially continuous phenomena by a logical sequence of gray (or color) tones that symbolize equal values. Isolines are often overlaid on top of an isopleth map to indicate threshold values.

## Methods

### Data sources

In February 2000, the Public Health Agency of Canada (PHAC) organized a National Steering Committee to develop a coordinated approach to respond to WNv [24]. As a result, a surveillance program was established to monitor WNv in humans, mosquitoes, birds and horses. The human and bird data utilized in this paper were obtained from this surveillance program for the years 2002 to 2005. The bird surveillance data including the number of birds tested and the numbers of WNv positive birds for each PHU were obtained from the Canadian Cooperative Wildlife Health Centre (CCWHC) WNv database. The human surveillance data, including the number of human WNv cases for each PHU were obtained from the Ontario Ministry of Health and Long Term Care (OMHLTC).

Estimates of the total population for each public health unit were calculated in 2001 for the Census of Canada and were obtained from Statistics Canada [25].

### Data collection

Dead birds were submitted for WNv diagnosis by both the general public and public health personnel as part of the National WNv surveillance program. The dead birds were tested for WNv infection by the CCWHC, provincial laboratories and the PHACs National Microbiology Laboratory in Winnipeg. In 2002 submitted dead birds were tested by the reverse transcriptase polymerase chain reaction (RT-PCR) test, which detected WNv ribonucleic acids (RNA) [26]. From 2003 to 2005 testing of submitted birds was carried out as a modified two-stage test. The first test was the oral VecTest™ (Medical Analysis Systems, Camarillo, CA), which was performed on oropharyngeal swabs from birds and detects WNv antigen. The sensitivity and specificity of the oral VecTest™ for crows collected in 2002 were 83.3% and 95.8%, respectively [27]. The second test, RT-PCR was used on only the positive birds that were the first bird in the PHU or major municipality of a PHU to be found positive. This sequential testing was performed in order to minimize false test positive results that would initiate unnecessary public health interventions. A suspected first positive dead bird in a PHU was defined as a positive WNv case if it tested positive using the oral VecTest™ as well as the RT-PCT test. After confirmation of WNv activity in a particular public health unit by RT-PCR, all positive oral VecTest™ results were assumed to be positive WNv cases.

Probable or confirmed human WNv cases were reported to local and provincial health authorities by health care providers, since the WNv infection is a reportable disease of humans in Canada [28]. Blood samples of individuals suspected to have symptoms of WNv infection were sent to the OMHLTC Central Public Health Laboratory (CPHL). Testing of the blood samples involved three tests in series. The first test was the IgM enzyme-linked immunosorbent assay (ELISA), which if positive was run again to rule out false positive results. These two tests could be followed by the plaque reduction neutralization test (PRNT) to confirm the diagnosis [29]. A human blood sample was defined as a positive WNv case if the sample tested positive on both the IgM ELISA tests (probable case) or if the sample tested positive on both the IgM ELISA tests and the PRNT (confirmed case).

### Study area

**Figure 1:**
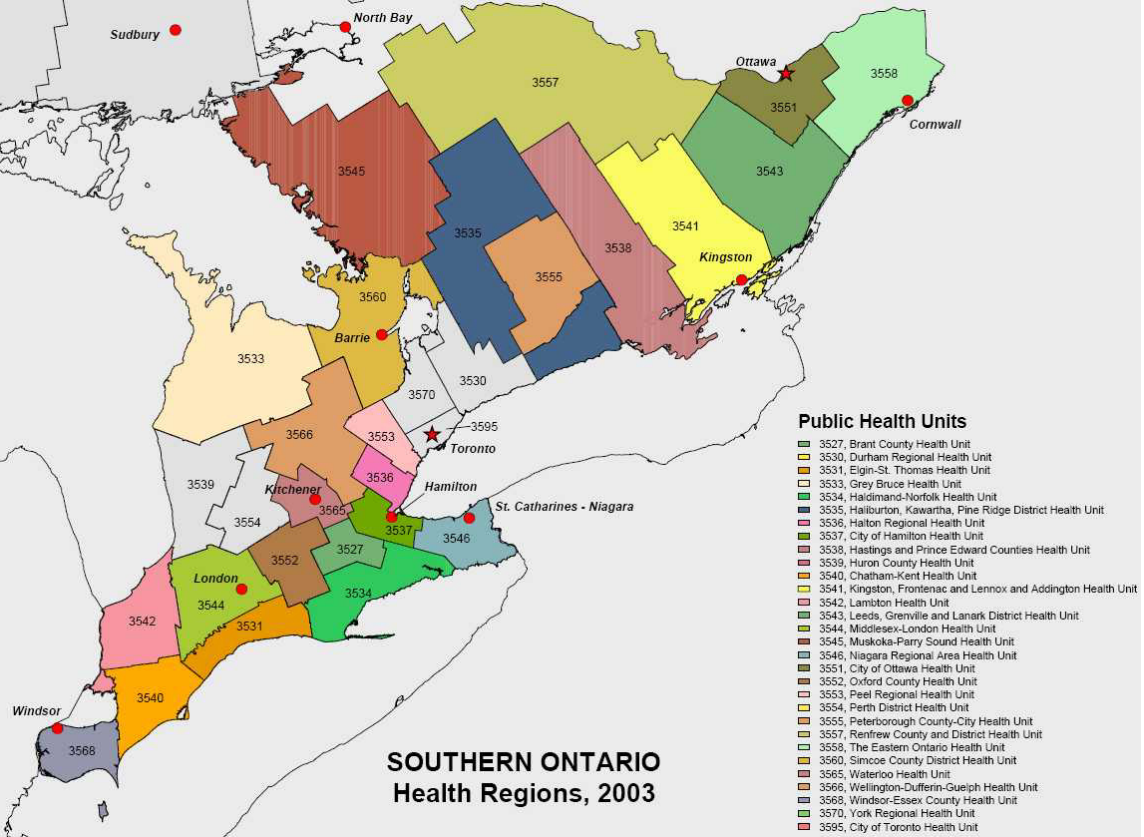
Description (names) and distribution of the Public Health Units of southern Ontario. The numbers represent the identifier code specific for each Public Health Unit.

The occurrence of WNv disease was investigated in the 30 PHUs of southern Ontario. PHUs are official health agencies that are responsible for administering health promotion and disease prevention programs [30]. PHUs were used as components of the study area in order for prevention and control methods to be implemented by epidemiologists or health care authorities of each unit. The PHU boundaries and the number of PHUs located in southern Ontario were consistent from 2002 to 2004. The names and distribution of the PHUs of southern Ontario are shown in Figure 1. In 2005 the Muskoka-Parry Sound PHU was dissolved. However for consistency of the results, the original 30 PHUs were used for the analyses of all four years.

### Statistical analyses

Crude PHU specific human incidence rates of WNv cases for the years 2002 to 2005 as well as the average annual incidence rates were calculated. The four annual incidence rates were calculated as the number of human WNv cases divided by the population totals for each PHU. The average incidence rates over the four years for each PHU were calculated by the sum of all four annual incidence rates divided by four. The four annual incidence rates and the average of the four years for each PHU were expressed as choropleth maps using the same incidence scale. This enables the reader to visualize the changes in crude incidence rates by region from year to year. The four annual mortality fractions and the average mortality fraction over the four years for birds were calculated in the same way, except, the bird WNv cases were divided by the total number of tested dead birds for each PHU. These were also expressed as choropleth maps.

There are often problems associated with mapping crude rates and fractions. For example, crude estimates of disease occurrence can be unreliable and highly variable as they are based on sample sizes or populations at risk, which can vary greatly from region to region [31]. In order to counter these problems, empirical Bayesian smoothing, also known as shrinkage estimation, was used to smooth the estimates.

Empirical Bayesian smoothing was performed to reduce the variance heterogeneity across the regional estimates [32]. The empirical Bayesian estimate is a weighted mean of the crude regional and global estimates. The weighting is determined by the variability of the estimates [33]. When the regional population is relatively large, the estimate is shrunk towards the global mean to a lesser extent and more weight is given to the regional estimate as there is more confidence in the precision of this estimate [31,32]. When the regional population is small, the shrinkage effect towards the global mean is stronger, as more weight is given to the global estimate, since there is less confidence in the precision of the regional estimate [31,32]. For empirical Bayesian smoothing, it was assumed that the bird data follow a binomial distribution and the human data a Poisson distribution.

The human and bird annual smoothed estimates, calculated by applying the empirical Bayesian smoothing method, are smoothed estimates of incidence rates and mortality fractions respectively, but for ease of reference, we refer to them collectively as smoothed risks. The average smoothed risks over the four years were calculated by the sum of all four smoothed annual risks divided by four. Choropleth maps as described above were then created with the empirically Bayesian smoothed risks for both the human and bird data. Again these choropleth maps were based on the same colour scale. Parallel boxplots of the crude rates and mortality fractions and the empirically Bayesian smoothed risks were created to show the shrinkage effect.

Choropleth maps are effective in showing the variation in regional data but are also known to have problems associated with them as outlined in the discussion. The geostatistical prediction method of kriging was used for isopleth mapping to overcome these problems. Kriging is an approach to interpolate or spatially predict regional data onto a continuous surface [34]. The kriging predictor is a weighted average that is calculated from the entire sample with weights depending on the semi-variogram [35]. The weights are constructed to give regional risk estimates more influence on the prediction the closer they are to the prediction sites and to downplay a cluster of points that contains largely redundant information [32]. The semi-variogram is a graphical representation of the variation between sampling points separated by a given distance and direction [31]. The semi-variogram was estimated by the weighted least squares estimation method [35]. Due to a limited number of PHUs, the maximum likelihood estimation method (MLE) was used for any years of data that could not be estimated by the weighted least squared estimation (WLSE) method. Risk maps for both the bird and human data were created to identify high risk areas and to investigate the potential relationship between the bird mortality fractions and the human rates. This was first done by visually checking for an overlap in the high risk areas on bird and human risk maps. The isopleth maps for all years investigated were created to have included the range of risk values for each particular year so that the bird high risk areas could be compared to the human high risk areas.

Furthermore, a Poisson regression analysis with an overdispersion parameter to control for clustering (i.e. spatial dependence) was used to model regional human WNv case counts as a function of the smoothed regional bird data and the spatial coordinates in the east and north direction [36]. A normal quantile-quantile (QQ) plot of scaled deviance residuals was plotted to evaluate the fit of the model and to identify public health units as potential outliers, i.e. disease clusters. The model is formulized as follows:

y_i_~Pois(exp{*β*_0_+*β*_1_x_1i_+........+*β*_*p*_x_pi_})

log(y_i_) = X*β *+ log(n_i_)

= *β*_0 _+ *β*_1_u_i _+ *β*_2_v_i _+ *β*_3_db_i _+ log(n_i_)

Var(y_i_) = *μ*_i _(1+*α*)

i = 1,..., 30 public health unit identifier

y_i _= number of cases in i-th public health unit

u_i _= easting coordinate of the i-th public health unit centre

v_i _= northing coordinate of the i-th public health unit centre

db_i _= empirical Bayesian smoothed risk for WNv infection within dead birds of the i-th public health unit

log(n_i_) = offset, log of population at risk of the i-th public health unit

*μ*_i _= X_i_*β*

*α *= overdispersion parameter

The presence of disease clustering and the location of disease clusters were investigated by the spatial scan test [37]. The spatial scan test is a likelihood ratio test, which uses circular scanning windows of various sizes and positions. By continuously changing the circle center and radius, the window scans the geographic area for potential localized clusters without incorporating prior assumptions about their size and location [38]. Circular search windows began with individual PHUs and expanded to include neighboring PHUs until a maximum of 50% of all dead birds investigated was reached, or – in the case of the human data – 50% of the total population at risk. A Bernoulli probability model was used to calculate the likelihood for the bird data, while the Poisson probability model was used with the human data. The spatial scan statistic tested the null hypothesis that the risk of the WNv disease within the window was equal to the risk outside the window, while the alternative hypothesis stated that there was an elevated risk for WNv infection within the windows as compared to outside the window. The *P*-value was obtained by Monte Carlo hypothesis testing, by comparing the rank of the maximum likelihood for the observed dataset to the maximum likelihoods of 999 simulated datasets [39]. Significant disease clusters (α = 0.05) were indicated on the choropleth maps of empirical Bayesian smoothed risks.

In order to investigate if the smoothed bird data helped to explain the human disease clusters the spatial scan test was repeated again for the human data. This time the smoothed bird data were added as a covariate. If the human clusters that were significant in the original spatial scan test (no covariate added) were no longer significant, then the covariate explained the human cluster or spatial distribution of human WNv disease.

All statistical analyses were carried out within R [40], except for the disease cluster analysis with the scan statistic, where SaTScan [39] was applied.

## Results

**Figure 2:**
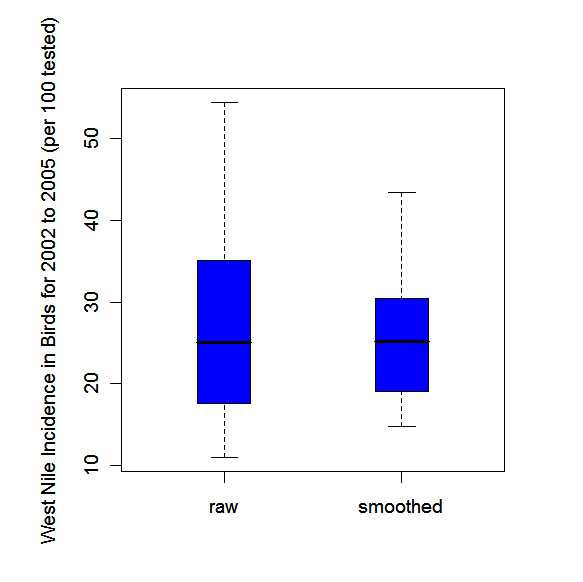
Parallel box plots for the raw annual WNv cause-specific mortality fractions per 100 birds tested (raw) and the corresponding empirical Bayesian smoothed estimates for the 30 public health units of southern Ontario, 2002–2005.

**Figure 3:**
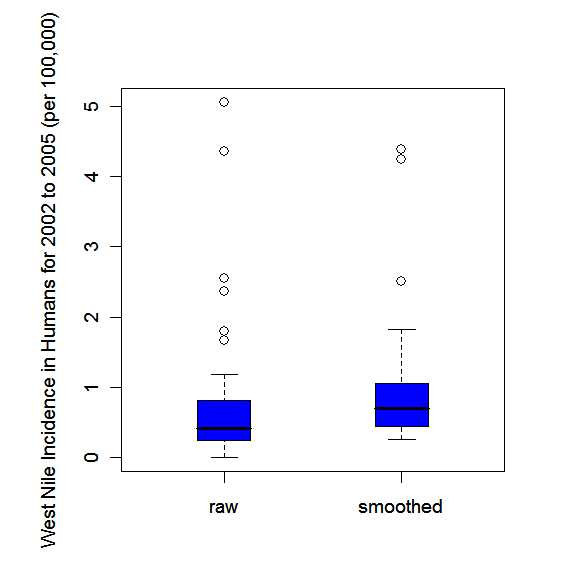
Parallel box plots for the raw annual human incidence rates of WNv disease per 100,000 population (raw) and the corresponding empirical Bayesian smoothed estimates for the 30 public health units of southern Ontario, 2002–2005.

Tables 1 and 2 give results for birds and humans, respectively. This includes crude rates or mortality fractions and empirical Bayesian smoothed estimates of WNv for all 30 PHUs in southern Ontario and for the years 2002 to 2005 as well as the four-year average. Mortality fractions in birds ranged from 0 to 83.3 cases per 100 dead birds tested with the highest values in the south of the study area. For humans the incidence rates ranged from 0 to 16.1 cases per 100,000 population. Again highest values were observed in southern PHUs. The empirical Bayesian smoothed estimates show less variation, ranging from 6.3 to 65.9 cases per 100 birds tested and in humans from 0.09 to 15.24 cases per 100,000 population. This shrinkage effect is also visualized by parallel boxplots of crude mortality fractions or rates and smoothed risk estimates in Figures 2 and 3, for dead birds and humans respectively.

**Table 1:** The 30 public health units in southern Ontario, the annual raw and Bayesian estimated mortality fractions of WNv per 100 birds tested for 2002 to 2005 as well as the average over the 4 years.

**Public Health Unit**		**Frequency/100 Birds** **Tested (2002)**		**Frequency/100 Birds** **Tested (2003)**		**Frequency/100 Birds** **Tested (2004)**		**Frequency/100 Birds** **Tested (2005)**		**Frequency/100 Birds** **Tested (Average)**	
**Number**	**Name**	**Raw**	**Bayesian**	**Raw**	**Bayesian**	**Raw**	**Bayesian**	**Raw**	**Bayesian**	**Raw**	**Bayesian**
3527	Brant	46.67	38.09	26.09	24.38	27.78	23.05	31.43	30.81	32.99	29.08
3530	Durham	10.20	16.08	12.24	14.41	15.38	16.28	11.59	12.69	12.35	14.86
3531	Elgin-St Thomas	70.00	44.68	30.77	25.98	25.00	21.53	25.00	25.70	37.69	29.47
3533	Bruce-Grey-Owen Sound	11.54	19.86	11.76	14.74	15.00	16.81	5.71	8.50	11.00	14.98
3534	Haldimand-Norfolk	83.33	43.83	56.25	39.79	66.67	33.86	11.11	16.94	54.34	33.61
3535	Haliburton-Kawartha-Pine Ridge	17.86	23.28	12.82	15.23	13.95	15.42	6.82	8.98	12.86	15.73
3536	Halton	48.48	42.22	48.39	39.86	27.08	24.66	48.78	46.23	43.18	38.24
3537	Hamilton-Wentworth	32.20	31.98	33.33	28.57	16.67	17.30	30.00	29.69	28.05	26.88
3538	Hastings and Prince Edward	46.15	39.90	12.00	14.19	10.45	12.30	6.38	8.47	18.75	18.71
3539	Huron	27.78	29.58	14.29	18.06	16.67	17.56	40.00	35.38	24.69	25.15
3540	Kent-Chatham	52.38	42.39	36.84	30.27	27.27	23.24	75.00	60.11	47.87	39.00
3541	Kingston-Frontenac-Lennox and Addington	55.00	43.48	19.05	20.13	0.00	6.32	12.50	14.54	21.64	21.12
3542	Lambton	22.86	25.82	43.75	33.24	21.74	20.33	50.00	44.17	34.59	30.89
3543	Leeds-Grenville-Lanark	31.25	31.28	9.30	12.44	2.78	8.35	5.56	10.41	12.22	15.62
3544	Middlesex-London	50.00	42.77	57.14	33.20	35.29	26.43	31.58	30.52	43.50	33.23
3545	Muskoka-Parry Sound	36.36	33.16	15.09	16.52	22.22	21.15	14.29	16.82	21.99	21.91
3546	Niagara	17.74	20.91	24.00	23.15	6.78	9.73	21.74	22.68	17.57	19.12
3551	Ottawa Carleton	20.00	25.49	22.73	22.47	15.79	16.76	11.76	13.19	17.57	19.48
3552	Oxford	28.00	29.42	29.41	25.85	19.23	18.99	26.67	26.68	25.83	25.24
3553	Peel	29.85	30.17	20.34	20.61	26.67	24.71	44.00	42.85	30.22	29.58
3554	Perth	50.00	39.87	29.41	25.85	19.23	18.99	41.67	37.06	35.08	30.44
3555	Peterborough	20.00	25.49	12.24	14.41	25.71	23.20	34.92	34.28	23.22	24.35
3557	Renfrew	33.33	32.09	10.34	12.62	20.45	19.91	3.45	7.08	16.89	17.93
3558	Eastern Ontario	33.33	32.09	14.29	16.82	10.71	13.97	12.50	16.07	17.71	19.74
3560	Simcoe	31.82	31.70	12.94	14.22	10.00	12.43	8.89	10.79	15.91	17.28
3565	Waterloo	32.50	32.12	16.67	18.31	21.05	19.85	31.43	30.81	25.41	25.27
3566	Wellington-Dufferin-Guelph	21.21	24.89	34.48	30.21	16.13	17.11	9.30	11.23	20.28	20.86
3568	Windsor-Essex	33.33	32.50	50.00	38.73	52.38	36.18	80.00	65.98	53.93	43.35
3570	York	25.81	27.89	22.50	22.29	14.47	15.33	40.74	39.48	25.88	26.24
3595	City of Toronto	47.37	39.35	43.59	37.64	36.73	31.60	61.67	58.81	47.34	41.85

**Table 2:** The 30 public health units in southern Ontario, the annual raw Bayesian estimated incidence rate of WNv per 100,000 population for 2002 to 2005 as well as the average over the 4 years.

**Public Health Unit**		**Frequency/100,000 Pop (2002)**		**Frequency/100,000 Pop (2003)**		**Frequency/100,000 Pop (2004)**		**Frequency/100,000 Pop (2005)**		**Frequency/100,000 Pop (Average)**	
**Number**	**Name**	**Raw**	**Bayesian**	**Raw**	**Bayesian**	**Raw**	**Bayesian**	**Raw**	**Bayesian**	**Raw**	**Bayesian**
3527	Brant	0.86	1.41	1.71	1.12	0.00	0.12	0.00	0.36	0.64	0.76
3530	Durham	0.60	0.76	0.00	0.28	0.00	0.10	0.00	0.12	0.15	0.32
3531	Elgin-St Thomas	0.00	0.98	0.00	0.64	1.25	0.18	0.00	0.46	0.31	0.56
3533	Bruce-Grey-Owen Sound	0.66	1.15	0.00	0.53	0.00	0.12	1.99	1.66	0.66	0.87
3534	Haldimand-Norfolk	2.91	3.09	0.00	0.60	0.00	0.13	0.00	0.40	0.73	1.05
3535	Haliburton-Kawartha-Pine Ridge	0.00	0.56	0.63	0.76	0.00	0.12	1.26	1.17	0.47	0.65
3536	Halton	16.10	15.24	0.00	0.34	0.00	0.11	1.34	1.28	4.36	4.24
3537	Hamilton-Wentworth	3.72	3.72	0.83	0.83	0.00	0.11	0.21	0.30	1.19	1.24
3538	Hastings and Prince Edward	0.00	0.60	0.00	0.53	0.00	0.12	0.00	0.31	0.00	0.39
3539	Huron	0.00	1.22	0.00	0.68	0.00	0.13	0.00	0.53	0.00	0.64
3540	Kent-Chatham	2.83	3.03	0.95	0.87	0.94	0.18	4.72	3.21	2.36	1.82
3541	Kingston-Frontenac-Lennox and Addington	0.00	0.52	0.58	0.73	0.00	0.12	0.00	0.28	0.14	0.42
3542	Lambton	1.59	1.99	0.00	0.56	0.00	0.12	1.59	1.37	0.80	1.01
3543	Leeds-Grenville-Lanark	0.00	0.57	0.00	0.52	0.00	0.12	0.00	0.30	0.00	0.38
3544	Middlesex-London	2.26	2.36	0.25	0.48	0.00	0.11	0.75	0.79	0.82	0.93
3545	Muskoka-Parry Sound	0.99	1.59	0.00	0.60	0.00	0.13	0.00	0.40	0.25	0.68
3546	Niagara	4.45	4.40	1.24	1.09	0.25	0.15	0.74	0.78	1.67	1.60
3551	Ottawa Carleton	0.00	0.13	0.52	0.60	0.13	0.13	0.39	0.44	0.26	0.33
3552	Oxford	0.00	0.84	0.00	0.61	0.00	0.13	1.02	1.00	0.26	0.64
3553	Peel	5.88	5.83	1.01	0.98	0.00	0.09	0.30	0.35	1.80	1.81
3554	Perth	1.38	2.04	0.00	0.65	0.00	0.13	0.00	0.48	0.35	0.83
3555	Peterborough	0.81	1.36	0.81	0.83	0.00	0.12	0.81	0.87	0.61	0.79
3557	Renfrew	0.00	0.86	1.05	0.90	0.00	0.13	0.00	0.42	0.26	0.58
3558	Eastern Ontario	0.00	0.50	0.00	0.49	0.00	0.12	1.10	1.06	0.27	0.54
3560	Simcoe	0.00	0.27	0.28	0.51	0.00	0.11	0.00	0.16	0.07	0.26
3565	Waterloo	0.69	0.88	0.23	0.45	0.00	0.11	0.23	0.33	0.29	0.44
3566	Wellington-Dufferin-Guelph	0.43	0.78	0.00	0.43	0.00	0.12	0.00	0.23	0.11	0.39
3568	Windsor-Essex	10.20	9.78	2.69	1.95	0.81	0.25	6.47	5.58	5.05	4.39
3570	York	1.52	1.60	0.28	0.42	0.14	0.13	0.69	0.71	0.65	0.72
3595	City of Toronto	6.63	6.60	1.79	1.70	0.24	0.20	1.55	1.53	2.55	2.51

**Figure 4:**
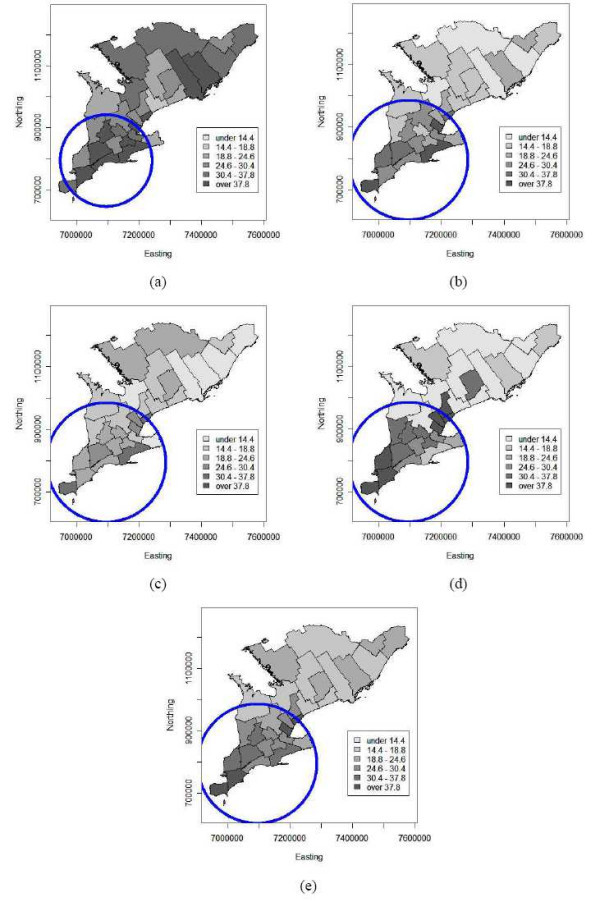
Choropleth maps of empirical Bayesian smoothed annual bird risk estimates of WNv disease per 100 birds tested for the 30 public health units of southern Ontario (a) 2002, (b) 2003, (c) 2004, (d) 2005 and (e) on average over 2002 to 2005. The circles indicate disease cluster locations as identified by the spatial scan statistic. Coordinates are in the Cartesian coordinate system (in meters).

Figures 4a) to 4e) show choropleth maps of the Bayesian smoothed risk estimates for birds for all four years and their average. The annual maps 2002 to 2005 have varying average risk levels and indicate highest WNv risks to occur mainly in the southern PHUs. Additionally the map of the average risk for the four-year period (Figure 4e) reveals the presence of a spatial trend with decreasing risk from south to north.

**Figure 5:**
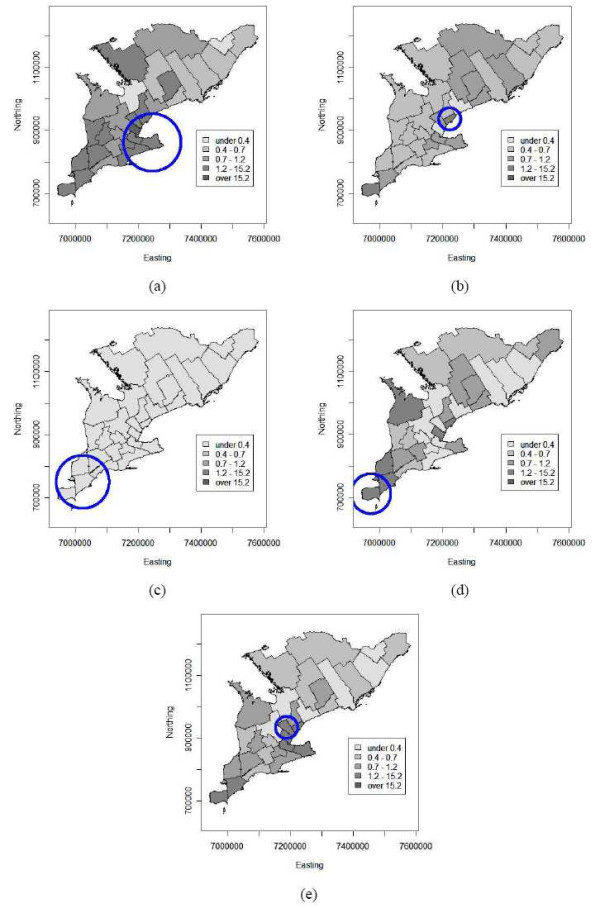
Choropleth maps of empirical Bayesian smoothed annual human risk estimates of WNv disease per 100,000 population for the 30 public health units of southern Ontario (a) 2002, (b) 2003, (c) 2004, (d) 2005 and (e) on average over 2002 to 2005. The circles indicate disease cluster locations as identified by the spatial scan statistic. Coordinates are in the Cartesian coordinate system (in meters).

Choropleth maps based on Bayesian smoothed human WNv risk are shown in Figures 5a) to 5e) for the years 2002 to 2005 and the corresponding four-year average, respectively. The annual maps show no clear spatial pattern of trend or clustering, except for 2002, when a potential cluster with a spike at the Halton PHU in the centre of the study area is indicated. This clustering seems to be consistent for all four years, since it is also indicated on the average map (Figure 5e). Perhaps most interesting is the smoothed risk map for 2004 (Figure 5c), as there were almost no human WNv cases reported and the map is constant over the entire study area at the lowest risk level.

In order to generate isopleth risk maps from smoothed data via kriging, spatial dependence was modeled by exponential semi-variograms without nugget effect. The models were fitted by weighted least squares to robust empirical semi-variograms for all of the bird data and for the first two years of human data. Semi-variogram models for the 2004, 2005 and the average human data were fitted by maximum likelihood estimation. The variogram clouds identified Halton PHU as an outlier within the 2002 and 2003 human data. Halton PHU was therefore excluded from the model fitting process. There were no outliers within the bird data. Table 3 shows the results of the range and sill values of the empirical semi-variogram for both the bird and human data for all years investigated. All empirical semi-variograms for the bird and human data over the years 2002 to 2005 leveled out and reached a sill. The semi-variograms based on the bird data indicated that the range of the semi-variogram increased from year to year. This showed that as the disease spread among birds in southern Ontario, the data were correlated over longer distances from year to year. For the human data, the semi-variogram parameters varied over the four years without any tendency.

**Table 3:** Semi-variogram estimates of the sill and range for the bird and human data from 2002 to 2005 as well as the average of the four years.

	**Sill**	**Range**
Bird 2002	82.29	81368.91
Bird 2003	82.34	132874.38
Bird 2004	56.34	245213.13
Bird 2005	355.07	335208.03
Bird Total	71.60	180000.00
Human 2002	3.03	184517.40
Human 2003	0.11	158159.40
Human 2004	0.001	101754.00
Human 2005	3.41	1270482.00
Human Total	1.04	112422.00

**Figure 6:**
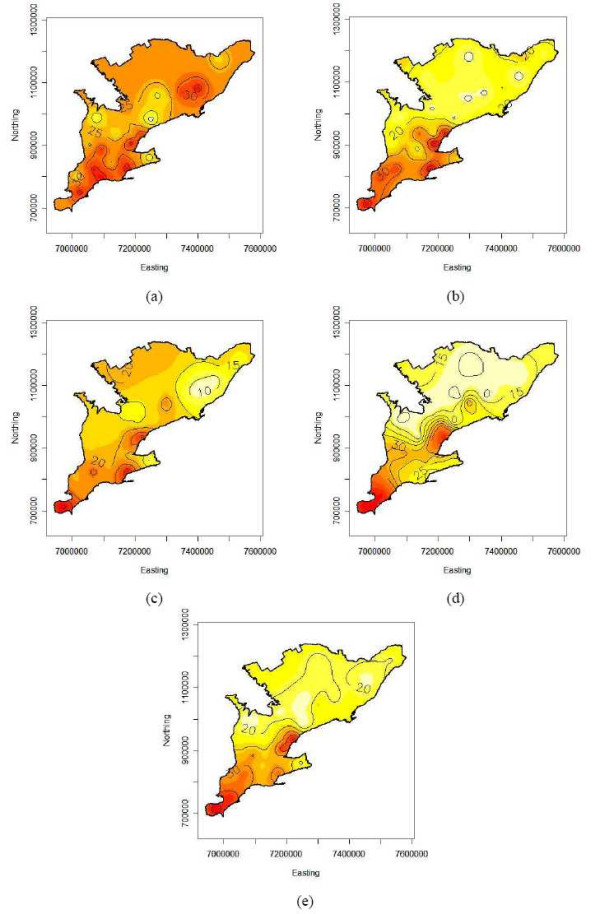
Isopleth maps from kriging the smoothed WNv bird risk estimates in southern Ontario (a) 2002, (b) 2003, (c) 2004, (d) 2005 and (e) on average over 2002 to 2005. Coordinates are in the Cartesian coordinate system (in meters).

**Figure 7:**
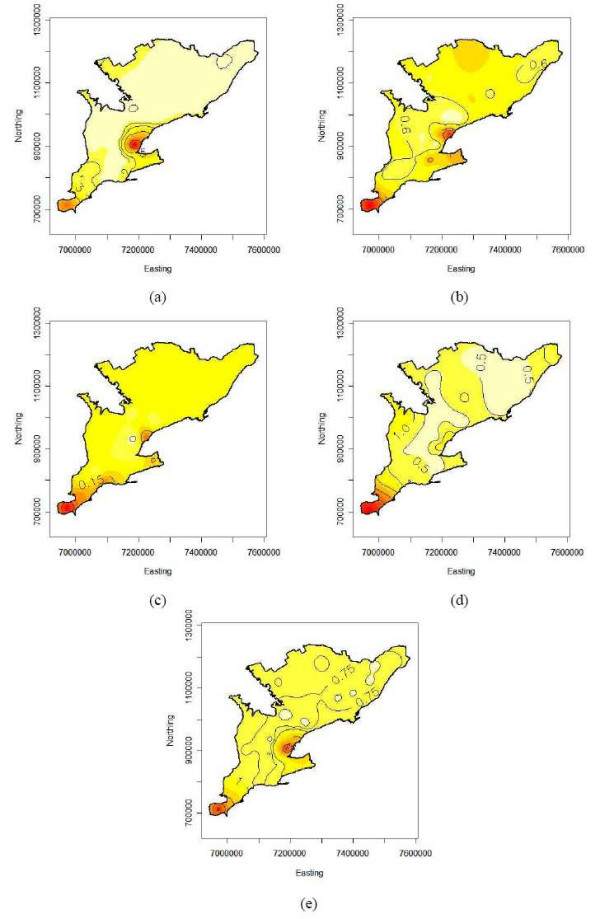
Isopleth maps from kriging the smoothed WNv human risk estimates in southern Ontario (a) 2002, (b) 2003, (c) 2004, (d) 2005 and (e) on average over 2002 to 2005. Coordinates are in the Cartesian coordinate system (in meters).

Figure panels 6 and 7 show isopleth risk maps for the five time periods resulting from ordinary kriging of the smoothed risk estimates derived from bird and human data, respectively. Figures 6a) to 6e) show several foci of elevated risk for birds in the southern PHUs of Ontario. From the average risk map for 2002 to 2005 (Figure 6e) it is seen that there are two major high risk areas in the centre and the south of the study area, i.e. around the Toronto-Peel-Halton and Windsor PHUs. Furthermore the spatial downwards trend in bird risk from south to north is clearly visible from this isopleth map. Similar results can be seen on the isopleth risk maps for humans. Although these maps do not exhibit a spatial trend, again the Toronto-Peel-Halton or Windsor PHUs stand out from the maps as high risk areas for human WNv illness (Figure 7).

The annual risks for southern Ontario were estimated by the intercept parameter of the spatial regression models used for kriging. The overall bird and human smoothed risks follow the same temporal pattern: steady decrease with a sharp increase in the last year. Explicitly, the overall smoothed risks were 31.9, 22.0, 19.2 and 25.2 deaths per 100 tested birds and 2.21, 0.76, 0.13 and 2.10 cases per 100,000 population for the years 2002 to 2005.

Poisson regression analysis indicated that the number of human cases had a significant relationship with the northing coordinate and the smoothed bird risk estimates (α = 0.05). The coefficients for the northing coordinate and the smoothed bird risk estimates were -7.714 *10^-6 ^and 0.036, respectively. The relative risk of human WNv disease was 0.46 times lower for a public health unit that was 100 km north of another public health unit (95% CI = 0.22–0.96), but it also increased by the factor 1.43 with every additional 10 positive birds per 100 tested (95% CI = 1.18–1.73). The QQ plot identified PHUs 3536, 3537, 3546, 3553 3568 and 3595 in 2002, PHUs 3553 and 3595 in 2004, and PHUs 3533, 3553 and 3595 in 2005 as outliers. Besides residuals of these outlying PHUs, the scaled deviance residuals for all other PHUs followed a normal distribution.

The scan test identified spatial disease clusters for the bird and human data for all time periods (α = 0.05) except for the 2004 human data (p = 0.057). The results of the spatial cluster analysis are summarized in Table 4 and visualized on the corresponding choropleth maps (Figures 4 and 5).

**Table 4:** Public health units included in most likely cluster, significance (p-value) of cluster, co-ordinates of the centre of the cluster and radius of the cluster for both the bird and human data from 2002 to 2005, as well as the average of the four years.

	**Public Health Unit included in Most Likely Cluster**	**(P-Value)**	**Co-ordinates (x,y)**	**Radius of Cluster**
Bird 2002	3531, 3544, 3552, 3542, 3540, 3534, 3527, 3554, 3565, 3539, 3537, 3536	0.011	7094370, 794983.35	147311.66
Bird 2003	3531, 3544, 3552, 3542, 3540, 3534, 3527, 3554, 3565, 3539, 3537, 3536, 3566, 3546, 3553, 3595	0.001	7094370, 794983.35	191155.89
Bird 2004	3531, 3544, 3552, 3542, 3540, 3534, 3527, 3554, 3565, 3539, 3537, 3536, 3566, 3546, 3553, 3595	0.001	7094370, 794983.35	191155.89
Bird 2005	3531, 3544, 3552, 3542, 3540, 3534, 3527, 3554, 3565, 3539, 3537, 3536, 3566, 3546, 3553, 3595	0.001	7094370, 794983.35	191155.89
Bird Total	3531, 3544, 3552, 3542, 3540, 3534, 3527, 3554, 3565, 3539, 3537, 3536, 3566, 3546, 3553, 3595	0.001	7094370, 794983.35	191155.89
Human 2002	3546, 3537, 3536, 3534, 3595, 3527, 3553	0.001	7243880, 862091,35	91430.61
Human 2003	3595, 3553	0.001	7222600, 936753,36	35169.79
Human 2004	3540, 3542, 3568, 3531	0.057	7022660, 750660.35	84304.62
Human 2005	3570, 3540	0.001	6972000, 712084.36	63198.43
Human Total	3536, 3595, 3553	0.001	7187530, 934088.36	35169.79

For the bird data, the scan test identified a locational stable WNv disease cluster in the south of the study area for each year as well as for the total over the four years. The 2002 significant cluster increased in radius to include four more public health units in 2003 and remained constant through 2005, see Figures 4a) to 4d).

Human spatial WNv clusters were located within the same areas where the WNv clusters among birds were identified. Although they were smaller in size, i.e. number of PHUs, and the location varied from 2002 to 2005. The human clusters are shown in Figures 5a-d for the years 2002, 2003, 2004 and 2005, respectively. The human WNv cluster for the four-year average is shown in Figure 5e). In addition, PHUs that were found to be outliers in the Poisson regression analysis were also identified as part of the disease cluster by the scan test.

The spatial scan test applied to the human data with the smoothed bird risk estimates as a covariate, resulted in no significant clusters for any of the four years or the average of the four years.

## Discussion

Using isopleth maps to display regional data has many advantages. For example isopleth mapping techniques overcome the problems or disadvantages of choropleth mapping. Choropleth maps can be misleading as the uneven shape and size of the different regions produce a visual bias [32]. For example, physically large areas that may be sparsely populated tend to dominate the perception of the map and may detract from smaller, sometimes more important regions, depending on the study [31]. Regional boundaries between census tracts also have problems associated with them. The risk of disease occurrence jumps artificially at boundaries from census tract to census tract and the risk is incorrectly assumed to be constant throughout each region or administrative area [34]. Isopleth mapping eliminates these problems since the risk distribution is mapped as a spatially continuous phenomenon that does not follow administrative boundaries.

The isopleth risk maps indicated that there were certain areas in southern Ontario where populations were at higher risk for acquiring WNv disease. The bird isopleth risk maps exhibited high risk areas that were relatively constant from year to year as the majority of the same PHUs were included in the high risk areas each year. The southern region of southern Ontario consistently contained the highest risk areas for birds over the entire study period. The high risk areas for humans did not exhibit quite as strong a pattern as the bird data, but there were a few PHUs that were high risk areas for the majority of the years investigated. These included the City of Toronto PHU, Kent-Chatham PHU and the Windsor-Essex PHU. By visual inspection of the risk maps for birds and humans for each year, it was found that there was an overlap of high risk areas. The overlaps occurred in the central-west and south-west areas of southern Ontario. Overlapping risk areas indicated that the high risk areas for birds can help to predict the occurrence of WNv disease in humans.

Human WNv disease clusters seemed to move in the south-west direction toward the most southern tip of southern Ontario. These clusters were still located within the boundaries of the bird WNv disease cluster. This movement of the human cluster may be an effect of the limited case numbers, which are furthermore not discriminated for the various types of WNv related illness.

WNv disease clusters as highlighted on the isopleth risk maps as well as identified by the spatial scan test, were meaningful in southern Ontario. These clusters cannot be regarded as chance clusters since they exist within both birds and humans for each year investigated. Neither can they be explained by perception bias (e.g. increased public awareness of WNv in urban areas compared to rural areas) because other urbanized centers do not show increased risk.

The analysis of disease clusters was an important part of this study. Kuldorff's spatial scan test used circular scanning windows to detect the potential cluster areas. A scan test that detects potential clusters based on non-circular windows may be more appropriate. For example, the flexible spatial scan statistic proposed by Tango and Takahashi [41], has the ability to detect noncircular clusters more accurately than circular clusters. Irregular shaped cluster should be identified in future research, but may require a sample size larger than 30 PHUs.

Both the scan statistic and the Poisson regression model indicated a relationship between the WNv cause-specific mortality fraction in birds and the human WNv incidence rates. The spatial scan test found that the human WNv disease clusters overlapped with those in birds for each year. The overlap of clusters indicated that an excess of bird cases has the potential to predict an excess of human cases. The scan test analysis of the human data that included the smoothed bird risk estimates as a covariate also supported this relationship. Once the smoothed bird risk estimates were added as a covariate the human clusters from the original scan test were no longer significant. The Poisson regression analysis indicated that the smoothed bird risk estimates explained the number of human WNv cases and indicated dependence between bird mortality fraction and human incidence rate data. The regression model further identified the northing coordinate as a significant predictor for the number of human cases, however other possible variables should be considered. The northing coordinate variable may be directly related to the variation in temperature from north to south. WNv amplification increases with temperature, thus explaining one reason that the southern regions of southern Ontario had the highest incidence rates and contained the WNv disease clusters. Other important variables related to temperature that would have had an impact on the occurrence of WNv disease, included weather and climate variables. In this analysis a climate (i.e. long period and large area average weather) variable would not have directly affected the spatial WNv risk pattern, as it would not have had an impact on the individual PHUs for a short time period. Climate change would have impacted the entire region of southern Ontario as a whole over a longer period of time. However, climate change would indirectly affect the occurrence of WNv illness in southern Ontario by creating extreme weather conditions. For example, weather conditions such as mild winters coupled with prolonged drought and heat waves, intensify the life cycle of WNv [42]. Rainfall is another variable to consider. A heavy rainfall will decrease the incidence of WNv as it flushes out sewers and drains that contained stagnant water, thus eliminating mosquito breeding sites. This study is analyzing annual data, which makes it difficult to incorporate weather events in a statistical model, e.g. heavy rainfall can prevent WNv disease for a few days but may further it when followed by a heat wave. Weather variables available at the PHU level should be included in a study that uses weekly data.

Although the tested dead bird data were useful for investigating the relationship between bird and human WNv occurrence, there were limitations or biases associated with the data. Passive surveillance is voluntary and is thus dependent on the public for finding and reporting dead birds. Surveillance can be affected by human related variables such as public awareness, public interest, media coverage and human density [28,43]. The PHUs of southern Ontario submitted birds at their discretion, therefore the analysis was highly dependent on non-homogenous sampling techniques. This may help to explain why the risk maps varied slightly from year to year. A more concise and standard sampling technique for all PHUs would be beneficial in the future. However, the surveillance data were adequate for the surveillance program's primary motivation of finding out when the WNv was active in each PHU.

The PHUs testing period (i.e. when the PHUs started and stopped testing dead birds) also manipulated the data. PHUs that did a lot of testing earlier in the season were not going to see as many cases as the PHUs who did the majority of their testing later in the year. This problem could be minimized by using tested dead bird data for the peak outbreak time as opposed to the whole year, thus increasing the stability of the risk pattern over time. The amount of testing also varied from year to year. For example, in 2005 the majority of PHUs carried testing further into the year as there were fewer cases in the beginning of the year.

In contrast, tracking dead bird sightings would have avoided delays associated with specimen collection and testing and would have allowed rapid recognition of trends in viral activity and the potential for occasional human cases or an outbreak [44]. Dead bird sighting data for southern Ontario were not used in the present analysis as not all public health units had sighting reporting systems in place. Furthermore, the available data were considered unreliable at the public health unit level because of sporadic and inconsistent sampling from year to year. In addition, the bird sighting data shared the human related issues mentioned above.

This study found a spatial relationship between the WNv cause-specific mortality fraction in birds and human WNv incidence rates, while other studies found a temporal relationship [20,38,45]. These studies have indicated that human WNv cases (onset of illness) will usually occur 2 to 5 weeks after bird WNv cases. By combining both the spatial and temporal relationship, prevention and control strategies can be implemented in certain high risk locations for specific high risk time periods. If public health authorities and health care authorities know where the high risk areas are for birds, then control measures such as application of adulticides or larvacides can be used to prevent human cases by reducing the vector population. Another important prevention and control method is public education. This initiative provides knowledge of the disease itself, how it is transmitted and how to prevent or reduce the risk of exposure. For example, a study by Loeb et al. [45] in the Halton region discovered that individuals who practiced at least two personal protective behavior traits such as wearing long sleeves, pants, insect repellent or eliminating standing water around one's home had a 50% reduction in the risk of infection.

## Conclusion

The risk pattern and locations of disease clusters among southern Ontario dead bird data were stable over time. The human WNv risk pattern and disease clusters were slightly more variable over time. The isopleth risk maps indicated that the WNv risk patterns for birds and humans overlapped. The spatial disease cluster analysis indicated that all human clusters fell within regions contained in the bird cluster. This indicates that WNv is established in southern Ontario.

There is a relationship between observed bird and human WNv cases as shown by the scan statistic and the Poisson regression model. Smoothed bird risk estimates can be used as indicators of high risk areas for humans. By knowing where high risk areas for birds and humans are, prevention and control methods can be intensified, thus mitigating human morbidity and mortality.

## Competing interests

The authors declare that they have no competing interests.

## Authors' contributions

HB contributed to the study design, performed the statistical analysis and wrote the manuscript. OB, JW and IKB contributed to the study design, interpretation of results and editing drafts of the manuscript. OB, JW and IKB also provided expertise and guidance throughout the research process.
